# Actinic Lichen Planus: Significance of Dermoscopic Assessment

**DOI:** 10.7759/cureus.35716

**Published:** 2023-03-03

**Authors:** Riddhima Singh, Sugat Jawade, Bhushan Madke

**Affiliations:** 1 Dermatology, Venereology and Leprosy, Jawaharlal Nehru Medical College, Datta Meghe Institute of Higher Education and Research, Wardha, IND

**Keywords:** dermoscopy, planus, lichen, actinic, rare

## Abstract

Actinic lichen planus (LP) is a rare variant of the already infrequent LP. LP is a chronic inflammatory skin disorder seen in 1-2% population of the world. The classical presentation is in the form of the four P's namely pruritic, purplish, polygonal, papules and plaques. On the contrary in this variant of actinic LP, although the lesions look similar in appearance they are characteristically distributed over the photo-exposed areas of the body like the face, extensors of the upper limbs, and dorsum of hands. Koebner's phenomenon which is characteristic of LP is absent. The commonest differentials that leave the clinician in a fix are usually discoid lupus erythematosus, granuloma annulare, and polymorphous light eruptions. A detailed clinical history followed by histopathological examination aids in the final diagnosis in such cases. In scenarios where the patient is not willing for a minor interventional procedure such as a punch biopsy, dermoscopic assessment comes to the rescue. Dermoscopy being an inexpensive, non-invasive, and minimal time-consuming procedure helps in the early diagnosis of a wide range of cutaneous disorders. Fine, reticulate white streaks over the surface of papules or plaques of LP known as "Wickham's striae" act as the diagnosis clincher for most cases of LP. The numerous variants of LP have consistent biopsy findings and the mainstay for treatment remains topical or systemic corticosteroids. We report this case of a 50-year-old female farmer that presented with multiple violaceous plaques on photo-exposed areas of the body owing to its rarity and use of dermoscopy in enabling a prompt diagnosis that helped improve the patient's quality of life.

## Introduction

Lichen planus (LP) is a known inflammatory skin disorder. It's seen in around 1-2% of the population worldwide. Most commonly encountered are classic LP lesions and are described using four P’s: purplish, pruritis, polygonal, and papules/plaques (flat-topped). Lesions often have a dry and glistening surface with a branny scale forming fine, white streaks, namely Wickham’s striae. Numerous variants in morphology and location occur as well, such as oral, nail, linear, hypertrophic, eruptive, LP pigmentosus, lichen planopilaris, and actinic lichen planus. But these are rare or occur less commonly compared to classic LP. A rarity in the variety of LP and an unusual presentation make prompt diagnosis and treatment challenging in dermatology clinics [1].

Actinic LP is a rare presentation of LP that affects areas exposed to the sun, like the forehead and extensor surfaces of upper extremities in dark-skinned individuals living in tropical or subtropical regions. There is often a higher predilection for the disorder during the summer months. In contrast with classical LP, features like pruritus, Koebner phenomenon, and the involvement of mucosal surfaces are not often seen. Nail involvement is almost always absent [2]. This is a case of a 50-year-old female farmer who presented with multiple violaceous plaques on photo-exposed areas of her body. She had previously gone to a local practitioner and was diagnosed with photoallergic dermatitis and came to our department upon no relief of her complaints. We could diagnose her promptly with actinic LP with the help of a dermoscopy.

## Case presentation

A 50-year-old female, a farmer by occupation, came to our outpatient department with chief complaints of multiple raised dark brown-purplish lesions on the lower back and both upper arms for three months. The lesions were not associated with itching or any other associated complaints. She had a history of previously being diagnosed with a case of sun allergy by a local practitioner and was given medications in the form of topical emollients.

**Figure 1:**
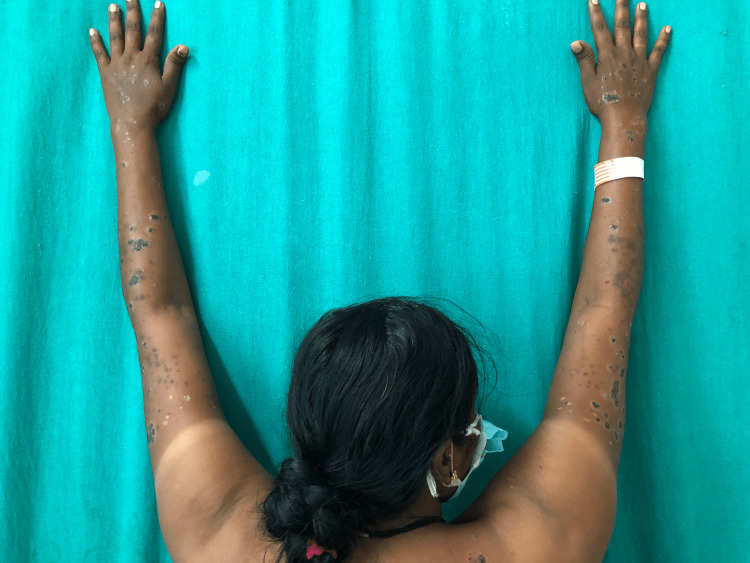
Symmetrical involvement of the extensor sun-exposed (distal 3/4th) areas of bilateral upper limbs.

**Figure 2:**
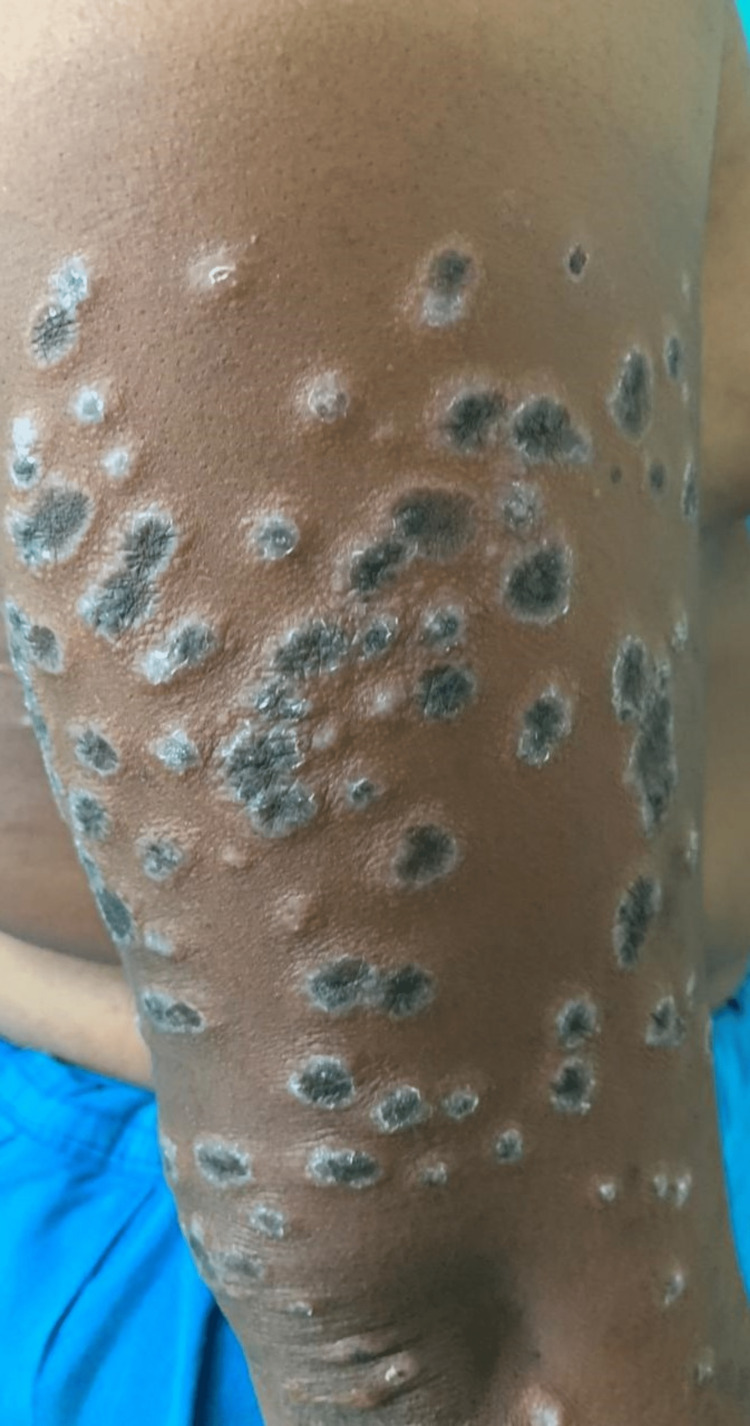
Multiple hyperpigmented plaques with distinctive hypopigmented borders on the right arm.

**Figure 3:**
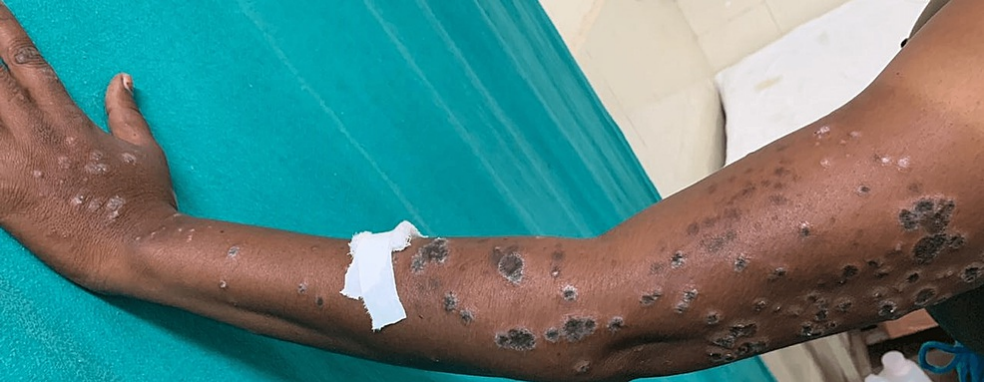
Symmetrical involvement of distal left upper limb (distal 3/4th) in the form of multiple violaceous and a few hyperpigmented plaques with a distinctive hypopigmented border.

**Figure 4:**
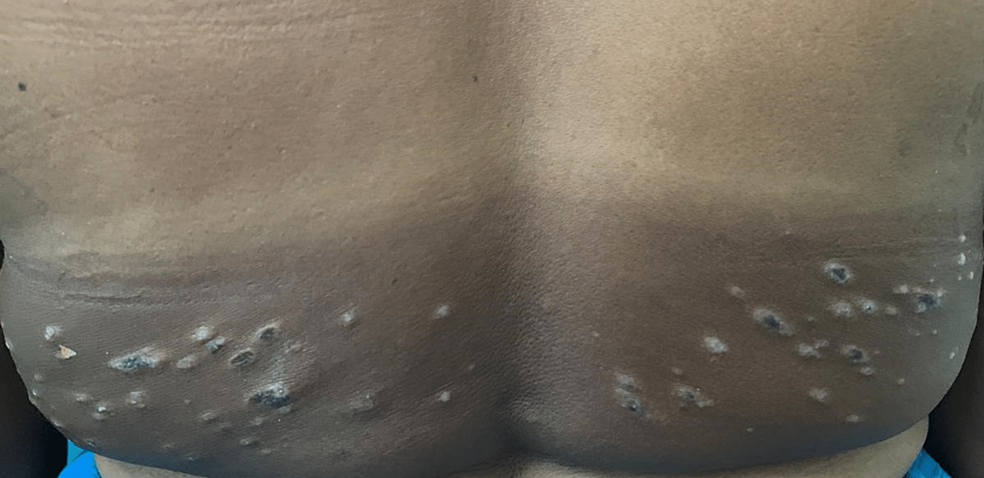
Involvement of the sun-exposed area of the lower back in the form of multiple violaceous plaques and a few papules with hypopigmented borders.

Detailed cutaneous examination revealed symmetrical involvement of bilateral upper limbs (distal 3/4th) in the form of multiple violaceous and some hyperpigmented plaques with distinctive hypopigmented borders. Similar lesions were also seen over the lower back (Figures 1-4). Oral mucosa and the nails were spared.

**Figure 5:**
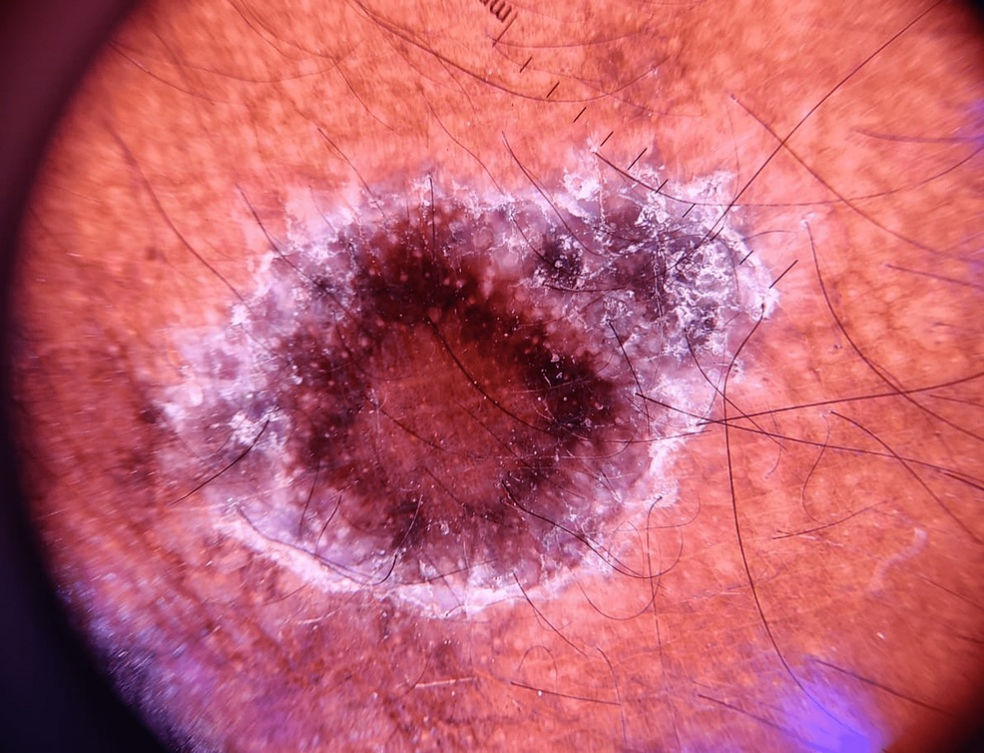
Dermoscopy image of one of the lesions showing fine, reticulate Wickham's striae over a violaceous background and diffuse brownish hyperpigmentation.

Dermoscopic assessment of lesions done using DermLite 3N (Dermlite LLC, San Juan Capistrano, California, USA) showed fine reticulate white lines, Wickham’s striae, diffuse brownish hyperpigmentation on a violaceous background (Figure 5). This further adds to our provisional diagnosis of actinic LP.

The patient's normal routine blood investigations were within normal limits. Histopathological examination (4 mm punch biopsy) from one of the lesions showed a lichenoid reaction pattern characterized by degeneration of the basal layer of the epidermis and a band-like lymphocytic infiltrate obscuring the dermo-epidermal junction. There was a prominent lymphocytic infiltrate and pigmentary incontinence. Our main differential diagnosis included discoid lupus erythematosus, sarcoidosis, and LP pigmentosus. The classical clinical picture of plaques with distribution over photo-exposed areas along with the above characteristic histopathological finding helped us in ruling out the differentials and arriving at a final diagnosis of actinic LP.

The patient has been prescribed oral prednisolone 40 mg once daily for a week, followed by tapering by 10 mg every week thereafter. The patient has also been advised of strict sun protection in the form of a hat/umbrella and fully covered limbs while working with a broad-spectrum sunscreen with Sun Protection Factor 30 all over the body and face.

## Discussion

LP is an inflammatory dermatological disorder with distinctive clinical and histopathological features. In addition to classic LP, many LP variants like oral LP, nail LP, linear, annular, hypertrophic, inverse, eruptive, bullous, ulcerative, LP pigmentosus, lichen planopilaris, vulvovaginal, actinic, LP-lupus erythematous overlap syndrome, and LP pemphigoides. Pruritic, polygonal, violaceous, flat-topped papules and plaques that are synonymous with classic LP are most commonly seen in clinical practice. The morphology and distribution differ largely depending on the type of LP but histopathological examination reveals consistency throughout all types [1].

Actinic LP is a rarely reported type of LP. Often documented using a variety of different terms like lichen planus subtropicus, lichen planus tropicus, and lichen planus actinicus. It is majorly present in young adults, and lesions are usually asymptomatic. It often erupts in spring or summer and involves sun-exposed areas, including the face [3]. The face is otherwise spared in the case of a classical variant of LP.

In clinics, actinic LP presents three types: annular, pigmented, and dyschromic. Out of these, the most frequently presenting form is the annular actinic LP which manifests as erythematous brown plaques that are annular in configuration. These lesions may or may not be associated with atrophy. Confirmation of diagnosis depends upon distribution over photo-exposed areas followed by a histological examination that reveals findings similar to those of classic LP [1].

The differential diagnoses of actinic LP that often leave clinicians in a fix include discoid lupus erythematosus, polymorphous light eruption, granuloma annulare, sarcoidosis, and LP pigmentosus, and these can be differentiated by careful history plus histopathologic findings [2]. In our case, previously misdiagnosed as a sun allergy by a local practitioner, performing bedside dermoscopy of the lesions came to our rescue, demonstrating Wickham's striae was our diagnosis clincher.

Dermoscopy of LP may be described in two phases. In the case of active LP, dermoscopic findings of structured white areas called Wickham striae have been reported in almost all studies. These correlate with epidermal proliferation in the form of wedge-shaped hypergranulosis and irregular acanthosis. Epidermal proliferation, in turn, leads to decreased visualization of blood vessels. Diffuse brown pigmentation in a few patients suggests epidermal pigmentation [4]. In the phase of the resolution, studies report no erythema or presence of blood vessels. There is also an absence of Wickham striae, although diffuse brown background with fine or coarse gray-blue dots is visualized in some patients, with brown to brown-black dots of various patterns present in all patients. Thus, Wickham striae are characteristically present in active lesions and disappear with treatment, but the pigment patterns resist treatment [4]. The etiopathogenesis has not been well documented, but numerous studies showed that lesions could be reproduced with ultraviolet radiation [2]. Histopathological findings are similar to those found in classical LP and show a linear band-like infiltrate (mostly lymphocytes), saw-toothed rete ridges, and dense hypergranulosis with orthokeratosis. The earliest feature seen in histopathology is degenerated keratinocytes, known as Civatte bodies [5].

In most scenarios, cutaneous LP tends to be self-limiting and lasts a few years. Therefore, most therapeutic modalities aim to relieve pruritus and fasten the resolution of lesions. Residual hyperpigmentation is very common. A wide number of treatments are available that include topical, intralesional, and systemic corticosteroids. They are indeed able to successfully reduce discomfort and inflammation. Although, owing to rebound and the high risk of relapse, a longer duration of corticosteroids is best avoided. Other options include hydroxychloroquine, metronidazole, azathioprine, systemic retinoids, dapsone, cyclophosphamide, interferon alpha-2b, cyclosporine, and tetracycline [5]. Strict sun protection should be advised to all patients of actinic LP in the form of protective full-sleeved clothing, equipment like a hat or an umbrella, and a broad spectrum sunscreen that should ideally be re-applied every three hours during peak solar radiation.

In our patient's scenario, systemic steroids had to be considered because of widespread involvement, causing morbidity to the patient. We prescribed her oral prednisolone 40 mg once a day for a week and planned a slow tapering by 10 mg every week thereafter. Along with the systemic immunosuppressant, strict sun protection was also advised. The patient was lost to follow-up because of covid-19 pandemic.

## Conclusions

Dermatological disorders have overlapping presentations and need years of expertise to be differentiated from each other. In settings where invasive procedures are unavailable or where the patient is not compliant, dermoscopy can be readily used to aid diagnosis, especially in inflammatory and pigmentary skin disorders. An early referral to a dermatologist may have prevented the misdiagnosis of this case and improved the patient's quality of life. Through this case, we report a rare entity of actinic LP and the significance of bedside dermoscopic assessment that enables making an early diagnosis of the same.
